# Epigenetic Mechanisms in CRSwNP: The Role of MicroRNAs as Potential Biomarkers and Therapeutic Targets

**DOI:** 10.3390/cimb47020114

**Published:** 2025-02-10

**Authors:** Alkmini Gatsounia, Georgios Schinas, Gerasimos Danielides, Katerina Grafanaki, Nicholas Mastronikolis, Constantinos Stathopoulos, Spyridon Lygeros

**Affiliations:** 1 Department of Otorhinolaryngology, Medical School, University of Patras, 26504 Patras, Greecenmastr@upatras.gr (N.M.); spyroslygeros@gmail.com (S.L.); 2 Medical School, University of Patras, 26504 Patras, Greece; georg.schinas@gmail.com; 3 Department of Dermatology-Venereology, School of Medicine, University of Patras, 26504 Patras, Greece; grafanaki@med.upatras.gr; 4 Department of Biochemistry, Medical School, University of Patras, 26504 Patras, Greece; cstath@med.upatras.gr

**Keywords:** chronic rhinosinusitis, nasal polyps, microRNAs, epigenomics, biomarkers

## Abstract

Chronic rhinosinusitis with nasal polyps (CRSwNP) is a prevalent inflammatory disease of the upper airway, contributing significantly to the global disease burden. CRSwNP is characterized by sustained and exaggerated inflammation, accompanied by marked changes in gene and protein expression regulated through intricate molecular mechanisms. MicroRNAs (miRNAs), small single-stranded RNA molecules that regulate gene expression at transcriptional and post-transcriptional levels, have emerged as pivotal players in CRSwNP pathophysiology. Dysregulated miRNA expression is implicated in numerous human diseases, including cancer, asthma, and inflammatory disorders, highlighting their broad clinical relevance. In CRSwNP, miRNAs influence important inflammatory pathways, including T2 immune responses and epithelial–mesenchymal transition (EMT), which leads to chronic inflammation and tissue remodeling. Profiling studies have identified specific miRNAs as potential biomarkers for disease severity, prognosis, and therapeutic response, offering a pathway to personalized medicine. Furthermore, advances in small extracellular vesicles (sEVs) and exosomes, which naturally transport miRNAs, provide innovative avenues for targeted miRNA delivery, minimizing systemic side effects. This review explores current knowledge on miRNA expression and function in CRSwNP, emphasizing their role in disease pathogenesis and their potential as biomarkers and therapeutic targets.

## 1. Introduction

Chronic rhinosinusitis (CRS) is a prevalent and debilitating health condition, affecting 5–12% of the global population and significantly impairing quality of life [1]. Among the phenotypes of CRS, chronic rhinosinusitis with nasal polyps (CRSwNP) represents a specific subset characterized by the formation of benign, edematous nasal polyps and a dominant type 2 helper T-cell (Th2) inflammatory response. CRSwNP affects 2.3–2.7% of the population and poses significant clinical challenges due to its chronic nature and resistance to conventional therapies [2,3]. Persistent symptoms severely impact patients’ productivity and quality of life.

Predisposing factors for CRS include lower airway diseases, such as asthma, chronic obstructive pulmonary disease (COPD), and bronchiectasis. Notably, asthma serves as an independent predictor for both CRS with and without nasal polyps, with a strong association observed in CRSwNP [2]. Smoking, encompassing both active and passive exposure, is also recognized as a significant risk factor for CRS, as the pollutants and toxins in tobacco contribute to oxidative stress and induce pro-inflammatory responses in the nasal mucosa [1]. Additionally, atopy is frequently observed in CRSwNP, with atopic patients demonstrating an increased risk of disease recurrence. Allergic rhinitis often coexists with CRSwNP, further complicating disease management and contributing to its clinical heterogeneity [1].

Anatomical variants that impede sinus drainage, obstructing the ostiomeatal complex, are additional predisposing factors that can contribute to the development of CRS. Furthermore, bacterial pathogens, particularly Staphylococcus aureus, play a critical role in the pathophysiology of CRS, with biofilm formation implicated in the persistence and severity of the disease [1]. These multifactorial contributors highlight the complexity of CRS pathogenesis, emphasizing the need for targeted diagnostic and therapeutic approaches tailored to individual patient profiles.

CRSwNP imposes a substantial burden, both in terms of patient morbidity and economic costs, due to recurrent symptoms, frequent medication use, and repeated surgical interventions. In severe cases, biologic therapies targeting specific immune pathways, such as IL-4 or IL-5, are employed. While these biologics offer hope for some patients, they are costly and not universally effective, highlighting the need for novel therapeutic approaches.

Epigenetics refers to the mechanisms by which genes interact with environmental factors to shape a phenotype. In the context of CRSwNP, exploring epigenetics aims to identify the specific genes and the mechanisms through which they respond to environmental triggers, ultimately driving inflammation and nasal polyp formation. Epigenetic mechanisms, including DNA methylation, histone modification, and regulation by non-coding RNAs, influence key processes such as inflammation, immune responses, and tissue remodeling. These modifications are highly dynamic, influenced by environmental factors, and often reversible, allowing cells to adapt to varying stimuli. Among these, microRNAs have emerged as particularly promising regulators in CRSwNP, playing crucial roles in modulating inflammation, epithelial dysfunction, and tissue remodeling.

This review delves into the emerging role of miRNAs in the pathogenesis of CRSwNP, emphasizing their influence on disease progression and their potential applications in diagnosis and treatment. A deeper understanding of miRNA regulatory mechanisms in CRSwNP is crucial for developing more personalized and effective treatment strategies, ultimately aiming to reduce the disease burden on patients and improve outcomes within healthcare systems.

## 2. Background on Epigenetics

Epigenetic regulation encompasses molecular mechanisms that influence gene activity and expression without altering the DNA sequence. These processes are dynamic, reversible, and shaped by environmental factors, allowing cells to adapt gene expression to changing conditions [4]. The field of epigenetics has garnered significant attention in recent years due to its potential to provide targeted treatments for a variety of diseases, including cancers, hematologic malignancies, and inflammatory conditions. Despite this progress, only a limited number of epigenetic drugs have been approved for clinical use, with most being utilized primarily for the treatment of cancers [5].

CRSwNP is a multifactorial condition shaped by the interplay of genetic predispositions, environmental triggers, and epigenetic modifications [1]. Studies indicate that first-degree relatives of CRSwNP patients are over four times more likely to develop the condition and have a significantly higher prevalence of nasal polyps than the general population [6,7]. This highlights the strong genetic component of the disease. Similarly, environmental factors such as allergens, pathogens, and pollutants drive epigenetic changes, altering gene expression without modifying the underlying DNA sequence [4]. This convergence of genetic and environmental influences through epigenetic mechanisms underpins the disease’s pathophysiology [8]. Advancing our understanding of these interactions holds promise for innovative therapeutic strategies and improved management of CRSwNP.

### 2.1. DNA Methylation

DNA methylation involves the addition of methyl groups to cytosine bases, primarily at CpG dinucleotides, often leading to gene repression when occurring in promoter regions. Abnormal DNA methylation patterns are strongly linked to various diseases, including cancers and airway inflammatory disorders such as bronchial asthma and allergic rhinitis [9,10]. Notably, nasal DNA methylation has emerged as a promising biomarker for asthma, allergic rhinitis, and airway inflammation, underlining its importance in understanding disease mechanisms and developing new therapeutic approaches [11]. In CRSwNP, hypomethylation of genes like IL-8 has been linked to increased pro-inflammatory cytokine expression, exacerbating inflammation and disease progression [12]. Similarly, hypermethylation of the TSLP and COL1A8 genes, associated with immune dysregulation and extracellular matrix remodeling, respectively, highlights their role in disease mechanisms [10].

### 2.2. Histone Modifications

Histone proteins, which package DNA into chromatin, are subject to chemical modifications such as acetylation, methylation, and phosphorylation. These modifications influence chromatin accessibility and, consequently, gene transcription. Histone acetylation generally promotes gene expression, while histone deacetylation suppresses it. In CRSwNP, histone deacetylase (HDAC) inhibitors have shown promise in reversing epithelial–mesenchymal transition (EMT) and mitigating inflammation, suggesting a potential therapeutic role for targeting histone modifications [13].

### 2.3. Alternative Polyadenylation (APA)

Alternative polyadenylation modifies the 3′ untranslated region (UTR) of mRNAs, influencing mRNA stability and translational efficiency. Dysregulated APA patterns have been observed in CRSwNP, suggesting their involvement in the chronic inflammatory state of the disease [10]. These alterations may affect the expression and function of key proteins involved in inflammation and tissue remodeling [4].

### 2.4. Non-Coding RNAs (ncRNAs)

MiRNAs are small RNA molecules (18–22 nucleotides) that suppress gene expression by targeting the 3′ untranslated regions of mRNAs. They are involved in various biologic processes, including cell proliferation, differentiation, and immune responses, and are implicated in the initiation and progression of several diseases. Emerging evidence implicates dysregulated miRNAs playing a central role in CRSwNP pathogenesis by modulating inflammatory pathways and tissue remodeling [14].

Among epigenetic mechanisms, non-coding RNAs, particularly microRNAs (miRNAs), have gained prominence for their unique ability to precisely regulate gene expression with high specificity at both transcriptional and post-transcriptional levels, simultaneously impacting multiple pathways. Unlike other epigenetic regulators, miRNAs are easily detectable in body fluids, making them ideal non-invasive biomarkers and promising therapeutic targets [15]. As such, miRNAs represent a critical focus of research for developing personalized therapeutic strategies to mitigate the disease burden and improve patient outcomes in CRSwNP.

## 3. Methodology

### 3.1. Search Strategy

This narrative review focuses on the role of microRNAs (miRNAs) in the pathogenesis and therapeutic potential of chronic rhinosinusitis with nasal polyps (CRSwNP). A systematic search of the PubMed database was conducted using the following search algorithm:

((“Sinusitis”[Mesh]) OR “Nasal Polyps”[Mesh]) AND “MicroRNAs”[Mesh]

The search aimed to capture studies that investigated the involvement of miRNAs in CRSwNP pathophysiology, as well as their potential use as biomarkers or therapeutic targets.

### 3.2. Inclusion and Exclusion Criteria

**Figure 1:**
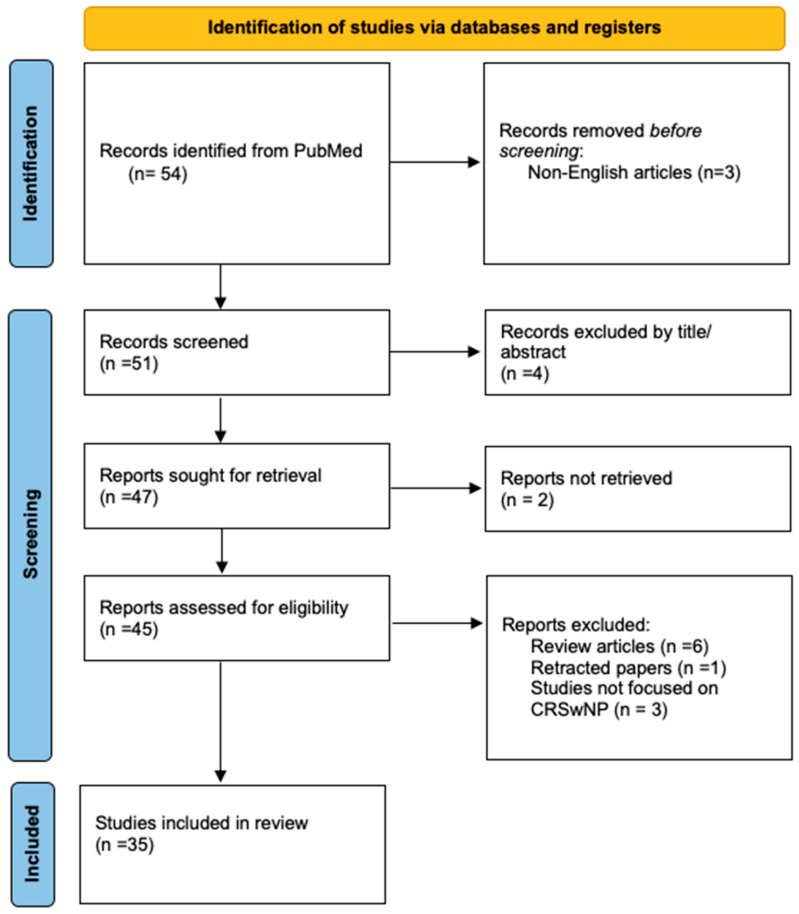
The Prisma 2020 flow diagram was used for the identification of the studies included in this review. No automation tools were used for the screening process.

Studies were included if they specifically addressed the role of miRNAs in the context of CRSwNP or sinusitis. Eligible studies investigated miRNA expression patterns, their target genes, associated molecular pathways, or their application as biomarkers or therapeutic interventions. Additionally, studies profiling miRNA expression in CRSwNP patients, including those employing ceRNA network analyses or exploring exosomal miRNA profiles, were included. Exclusions were applied to studies focusing solely on other epigenetic mechanisms, such as DNA methylation, or those that did not explicitly include CRSwNP patients or included other forms of sinusitis without distinguishing. Non-peer-reviewed articles, opinion pieces, review articles, and retracted articles were also omitted. The PRISMA flow diagram, presented below, provides a visual summary of the study selection process (Figure 1).

### 3.3. Data Extraction and Presentation

The review focused on extracting data relevant to miRNA profiles, including their identity, expression patterns (e.g., upregulated or downregulated), and the biological pathways they regulate. Functional roles of miRNAs in inflammation, immune responses, tissue remodeling, and their modulation by specific therapeutic agents (e.g., miRNA mimics, inhibitors, or other targeted therapies) were documented when available. Additionally, information extracted from studies profiling miRNA expression in CRSwNP patients included details on methodologies, key findings, and potential clinical implications and applications. The extracted information is organized into structured summary tables, one for studies investigating specific miRNAs and another for studies focusing

## 4. The Role of miRNAs in the Pathophysiology of CRSwNP

CRSwNP is mainly characterized by type 2 helper T-cell (Th2) dominant immune response, elevated levels of cytokines IL-4, IL-5, and IL- 13, accompanied by changes in IL-25, IL-33, TSLP, and IgE levels, leading to prolonged inflammation and tissue remodeling [16]. In addition, it is associated with elevated levels of eosinophils, innate lymphoid cells (ILC2), macrophages, and mast cells [17]. These events lead to epithelial cell alterations, epithelial–mesenchymal transformation, goblet cell hyperplasia, extracellular matrix degradation, fibrin deposition, tissue edema, and, finally, nasal polyp formation.

In CRSwNP, specific miRNAs have been identified that correlate with inflammatory and anti-inflammatory signaling pathways in CRSwNPs. These epigenetic alternations collectively contribute to the complex pathophysiology of CRSwNP. They highlight the interplay between environmental triggers and gene regulation, offering promising avenues for the development of targeted therapies and novel biomarkers to manage this burdensome condition.

### 4.1. Epithelial–Mesenchymal Transition (EMT) in CRSwNP

Epithelial–mesenchymal transition (EMT) is a critical process in tissue remodeling and nasal polyp formation in CRSwNP, driven by inflammatory and molecular signals, particularly TGF-β1, a well-known EMT inducer. TGF-β1 regulates multiple miRNAs that modulate EMT and tissue remodeling. For instance, TGF-β1 upregulates miR-182, which promotes EMT by facilitating epithelial cell transition to myofibroblasts. Inhibition of miR-182 reverses TGF-β1-induced EMT, positioning miR-182 as a potential therapeutic target [18]. Similarly, miR-21 plays a multifunctional role, facilitating EMT through the TGF-β1-miR-21-PTEN-Akt axis and promoting epithelial proliferation and survival via the EGF/HIF-1α/miR-21/AQP4 axis [19,20]. Another TGF-β1-driven pathway, the TGF-β1/miR-29b/HSP47 axis, contributes to collagen deposition and EMT, exacerbating tissue remodeling in CRSwNP [21]. The upregulation of miR-155-5p in CRSwNP plays a pivotal role in driving EMT and tissue remodeling through its interaction with TGF-β1 and sirtuin 1 (SIRT1) [22].

Small extracellular vesicles (sEVs) represent a key mechanism through which miRNAs regulate epithelial–mesenchymal transition (EMT). These vesicles, secreted by cells into the extracellular environment, play a critical role in intercellular communication by transporting biomolecules such as proteins, lipids, mRNAs, and microRNAs. sEVs enriched with miR-375-3p have been shown to promote EMT by targeting the KH domain containing RNA binding (QKI), a known suppressor of EMT, thereby facilitating this pathological process [23].

Downregulated miRNAs also influence EMT. MiR-30a-5p suppression promotes EMT, increasing inflammation and tissue remodeling [24]. Similarly, miR-18a activates the PI3K/AKT pathway by targeting PTEN, driving EMT and chronic inflammation [25]. Furthermore, the downregulation of miR-200a-3p leads to increased expression of ZEB1, a key EMT regulator, while promoting inflammation and tissue remodeling via the ERK/p38 and MAPK pathways [26].

### 4.2. Th2 Inflammation and Immune Dysregulation

Th2 inflammation, characterized by elevated cytokines such as IL-4, IL-5, and IL-13, is a hallmark of CRSwNP. Several miRNAs play pivotal roles in modulating Th2 inflammation. MiR-155 amplifies inflammation by promoting COX2 expression and suppressing SOCS1, leading to the release of cytokines such as TNF-α, IL-1, IL-4, and IL-5 [21]. Similarly, miR-125b enhances Th2 inflammation in eosinophilic CRSwNP by increasing IL-5 levels via the type I interferon (IFN) signaling pathway, contributing to eosinophil infiltration [27,28]. In addition to its role in EMT, miR-21 exhibits anti-inflammatory effects by increasing IL-10 levels and modulating the PDCD4/NF-κB pathway, demonstrating its dual role in regulating inflammation and tissue remodeling in CRSwNP [29]. Another contributor, miR-21-5p, upregulates IL-33 via the GLP-1R/IL-33 axis, promoting Th2-skewed inflammation and eosinophilia [30].

In contrast, some miRNAs demonstrate anti-inflammatory roles by suppressing Th2 inflammation, while others exacerbate inflammatory pathways in CRSwNP. For example, the downregulation of let-7a-5p in CRSwNP enhances the expression of pro-inflammatory cytokines TNF-α, IL-1β, and IL-6 through the Ras-MAPK pathway, thereby amplifying inflammation. Restoring let-7a-5p levels has been proposed as a potential therapeutic strategy for mitigating inflammation [31]. On the other hand, certain miRNAs actively promote inflammation. In eosinophilic chronic rhinosinusitis with nasal polyps (eCRSwNP), miR-155 is upregulated, driving inflammation by downregulating the anti-inflammatory mediator SOCS1 and upregulating the inflammatory mediator COX2 via the NF-κB pathway [32]. Additionally, miR-205-5p is significantly upregulated in CRSwNP patients, and its expression positively correlates with IL-5 levels, eosinophil counts, and a stronger Type 2 (Th2) inflammatory response [33]. These examples highlight the dual roles of miRNAs in both amplifying and suppressing inflammation, particularly in Th2-dominant CRSwNP, and suggest therapeutic opportunities to restore immune balance.

### 4.3. Cytokine Regulation and Immune Balance

Cytokine signaling plays a central role in CRSwNP inflammation and remodeling, with miRNAs acting as key regulators. For instance, miR-19a suppresses IL-10 production in peripheral dendritic cells, exacerbating inflammation and impairing immune regulation [34]. MiR-150-5p also modulates immune balance by suppressing EGR2, leading to increased Th17 activity and elevated IL-17 levels, further fueling inflammation [35].

Another example is the miR-4492/IL-10 axis, where downregulated miR-4492 correlates with increased IL-10 expression, suggesting its involvement in cytokine regulation through the Jak/STAT pathway [36]. Downregulation of miR-124 contributes to elevated AHR levels in CRSwNP, as miR-124 typically inhibits AHR by binding to its 3′ untranslated region [37]. This loss of regulation exacerbates inflammation in nasal polyps, highlighting the miR-124/AHR axis as a critical factor in the disease’s inflammatory response. Upregulation of both MiR-142-3p and TNF-a enhances inflammation in CRSwNPs through the LPS-TLR- TNF-α signaling pathway [38].

These studies highlight the dual roles of miRNAs in cytokine modulation and their potential as therapeutic targets for restoring immune balance in CRSwNP.

### 4.4. Vascular Remodeling and Structural Changes

Vascular remodeling and epithelial barrier dysfunction are hallmark features of CRSwNP, heavily influenced by miRNAs. MiR-22-3p, carried within exosomes, downregulates VE-cadherin, increasing vascular permeability and contributing to tissue edema and nasal polyp formation [39]. Exosomes, as membrane-bound microvesicles, play a crucial role in intercellular signaling by delivering miRNAs, mRNAs, and proteins to target cells via body fluids, thus significantly impacting CRSwNP pathophysiology [40].

Additionally, miR-214 upregulates STAT3/GDF15 and SIRT1 pathways, promoting mucus overproduction and LPS-mediated inflammation [41]. The downregulation of miR-29b-3p leads to α-tubulin deacetylation by increasing the number of matrix metalloproteinase 9 (MMP-9)-integrin β1 complexes in CRSwNPs, promoting polyp creation [42]. Downregulated miR-34 and miR-449 impair ciliogenesis by failing to suppress NOTCH1 and regulate ciliogenesis-associated genes [43].

Moreover, miR-125b is significantly upregulated in nasal polyps. Its role in activating the Wnt/β-catenin signaling pathway is triggered by Specificity protein 1 (Sp1), a transcription factor that upregulates miR-125b expression [44]. This activation results in increased inflammation, fibrin deposition, and the release of inflammatory cytokines, that contribute to the development and progression of nasal polyps in CRSwNPs. These findings suggest that miRNAs affecting vascular integrity and epithelial function play a pivotal role in disease progression.

An overview of miRNAs and their associated inflammatory pathways is provided in Table 1.

## 5. Decoding Molecular Pathways in CRSwNP: The Role of miRNA Profiling and Beyond

MicroRNA (miRNA) profiling has become a pivotal tool for characterizing the molecular basis of chronic rhinosinusitis with nasal polyps (CRSwNP). By systematically cataloging miRNA expression patterns in nasal tissues or peripheral blood cells, profiling paves the way for the identification of key pathways of disease progression. This technique also aids in stratifying patients into phenotypic subgroups based on molecular signatures, laying the groundwork for personalized therapeutic approaches. Distinct from traditional profiling approaches, which focus on the direct analysis of miRNA expression, advanced methodologies such as competitive endogenous RNA (ceRNA) network analysis and exosomal miRNA profiling offer additional layers of insight. Such efforts provide not only mechanistic insights but also point towards novel therapeutic targets. Together, all three existing approaches expand the scope of miRNA research and could hopefully bridge molecular findings with clinical applications. An overview of miRNAs profiling studies in CRSwNP is provided in Table 2.

### 5.1. Subtype-Specific Insights from miRNA Profiling

Profiling studies in CRSwNP have provided insights into subtype-specific molecular mechanisms. A study analyzing miRNA profiles in CRSwNP subtypes, eosinophilic (ECRSwNP) and non-eosinophilic (non-ECRSwNP) variants, in particular, has shed light on the distinct pathophysiological aspects implicated [46]. MiRNA families such as miR-154, miR-221, and miR-223 were implicated in both subtypes, while let-7 and miR-34/449 were identified as being specifically associated with non-ECRSwNP. Pathway analyses revealed that type 2 inflammation, exemplified by eosinophil migration and chemotaxis, dominated in ECRSwNP, while type 1 and type 3 inflammation, characterized by neutrophil activation and regulation of interferon-gamma production, were predominant in non-ECRSwNP. Moreover, another study highlighted the role of miRNA expression in mature dendritic cells (DCs) from CRS patients, identifying 31 differentially expressed miRNAs, including subgroup-specific patterns [47]. Specifically, miR-1290 was upregulated in CRSwNP but downregulated in CRSsNP, underscoring distinct regulatory roles across subtypes. Shared miRNAs such as miR-125b-5p were shown to regulate both epithelial and peripheral immune functions, linking epithelial changes with systemic immune dysregulation. Notably, miR-126-3p was implicated in regulating Th2 function, while miR-708-5p played a significant role in airway inflammation and could thus serve as promising candidates for therapeutic intervention. In another profiling study, miR-181b has been identified as a regulator of NF-κB signaling and a potential therapeutic target, while miR-125b and miR-155 hold promise as biomarkers for distinguishing CRS subtypes [48]. Finally, a similar study identified miRNAs linked to mucin-type O-glycan biosynthesis, such as miR-210-5p, introducing a less-reviewed pathway associated with excessive mucus production in CRSwNP, while downregulated miRNAs, including miR-32-3p, targeting TGF-β and MAPK signaling were reportedly linked in tissue remodeling and inflammation [49].

### 5.2. Regulatory Networks: ceRNA Mechanisms in CRSwNP

Beyond profiling, miRNA research in CRSwNP has advanced through the construction of competitive endogenous RNA (ceRNA) networks. A ceRNA network constitutes a regulatory mechanism where long non-coding RNAs (lncRNAs) act as “sponges” for microRNAs (miRNAs), thereby preventing miRNAs from binding to their target messenger RNAs (mRNAs). This interaction creates a complex lncRNA-miRNA-mRNA network that can influence gene expression and thereby provide insights into the underlying pathophysiology. A study employing such an analysis pinpointed the MIAT/miR-125a/IRF4 axis as a central player in immune cell infiltration and inflammatory responses [50]. This axis has demonstrated a strong diagnostic value (AUC = 0.944) and represents a promising target for therapeutic interventions aimed at mitigating eosinophil-driven inflammation and tissue remodeling. Other key regulatory elements uncovered by similar studies, such as CFI in the complement cascade and MSC-AS1 in chemokine signaling, further characterize the inflammatory pathways contributing to CRSwNP pathogenesis and may pose as future targets for intervention [51].

### 5.3. Exosomal miRNA Profiling: Systemic Insights and Therapeutic Potential

Exosomal miRNA profiling offers a distinct yet complementary perspective by capturing miRNAs in circulation rather than localized tissue. Exosomes act as carriers of miRNAs, enabling long-distance regulation of gene expression, which may systemically influence inflammation and tissue remodeling in CRSwNP [14]. A novel study of this kind identified the differential expression of 159 exosomal miRNAs, including 93 upregulated and 66 downregulated miRNAs, which were linked to pathways regulating epithelial–mesenchymal transition (EMT), mucosal remodeling, and chronic inflammation [52]. Functional predictions associated these miRNAs with key signaling pathways, such as Wnt/β-catenin and Hippo, which are involved in tissue remodeling in CRSwNP. These findings set the basis for exosomal miRNAs that could be engineered to deliver therapeutic payloads, targeting the aforementioned pathways, thereby offering a novel treatment platform. Furthermore, the non-invasive nature of plasma exosomal miRNA profiling positions it as a promising biomarker for diagnosing CRSwNP, monitoring disease progression, or possibly evaluating a therapeutic response. Future research should validate these findings in larger cohorts and explore their clinical utility as well as their potential applications.

**Table 2:** Summary of miRNA profiling studies in CRSwNP, key findings, and potential clinical applications.

Study	Methodology	Findings	Clinical Implications
Xuan et al., 2019 [49]	miRNA profiling using miRCURY™ LNA Array in nasal mucosa tissues of CRSwNP patients (n = 19) and healthy controls (n = 10)	Identified 5 upregulated miRNAs (e.g., miR-210-5p) and 19 downregulated miRNAs (e.g., miR-32-3p); pathways regulated include mucin-type O-glycan biosynthesis, TGF-β signaling, TRP channels, and MAPK signaling	The upregulation of miRNAs linked to mucin-type O-glycan biosynthesis suggests a role in excessive mucus production in CRSwNP, while the downregulation of miRNAs targeting TGF-β and MAPK signaling pathways highlights their involvement in tissue remodeling and inflammation. These miRNAs may serve as targets for modulating these pathways in future therapeutic approaches
Chen et al., 2022 [50]	Constructed a ceRNA network using bioinformatics and experimental validation; analyzed lncRNA, miRNA, and mRNA expression profiles from Gene Expression Omnibus datasets (42 CRSwNP tissues compared with 28 control samples)	Identified 565 DE-lncRNAs, 23 DE-miRNAs, and 1799 DE-mRNAs; constructed a ceRNA network centered on the MIAT/miR-125a/IRF4 axis; IRF4 was linked to immune cell infiltration, including eosinophils and M2 macrophages; MIAT and IRF4 correlated positively with dendritic cells and M2 macrophages	The MIAT/miR-125a/IRF4 axis plays a critical role in CRSwNP pathogenesis by regulating immune cell infiltration and inflammatory responses. IRF4 and MIAT show high predictive value for CRSwNP diagnosis (AUC = 0.944), offering potential as diagnostic biomarkers and therapeutic targets. Interventions targeting this axis may mitigate eosinophil-driven inflammation and tissue remodeling in CRSwNP
Li and Liu, 2022 [51]	Profiling of mRNAs, miRNAs, and lncRNAs; construction of ceRNA network linking miRNA–mRNA–lncRNA interactions in eosinophilic CRSwNP (12 eosinophilic CRSwNP nasal polyp samples and 12 normal control nasal samples were included)	Identified 358 differentially expressed miRNAs (DEmiRs), 964 mRNAs (DEmRs), and 15 lncRNAs (DElncRs); key pathways enriched include cytokine–cytokine receptor interaction, chemokine signaling, complement cascades, and S. aureus infection pathways. CFI and MIAT were highlighted as hub elements in ceRNA networks	Identified miRNA–mRNA–lncRNA interactions reveal potential mechanisms behind inflammation, eosinophil infiltration, and NP formation. Specific hubs like MSC-AS1 (broad regulation) and CFI-associated networks suggest targets for future therapeutic interventions targeting ceRNA dynamics in eosinophilic CRSwNP.
Xia et al., 2015 [48]	RT-qPCR analysis of 7 inflammation-associated miRNAs (miR-181b, miR-26b, miR-155, miR-146a, miR-125b, miR-124, miR-92a) in 40 CRS patients and 5 controls	miR-125b, miR-155, and miR-146a were upregulated in CRS and NP; miR-92a, miR-26b, and miR-181b were downregulated. In CRS without NP, miR-125b and miR-155 were significantly upregulated, while miR-92a, miR-26b, and miR-181b were downregulated. miR-124 showed no significant change	miR-181b, as a regulator of NF-κB signaling via importin-β3, emerges as a potential therapeutic target. miR-125b and miR-155 could serve as biomarkers for distinguishing CRS subtypes, while miR-92a and miR-26b are associated with decreased inflammation
Ma et al., 2015 [47]	miRNA microarray analysis of mature dendritic cells (DCs) from peripheral blood of CRS patients (n = 30) and controls (n = 7); RT-qPCR validation of selected miRNAs	Identified 31 differentially expressed miRNAs in CRS patients, with miR-125b-5p, miR-210-3p, and miR-150-5p upregulated, and miR-708-5p and miR-126-3p downregulated. Subgroup-specific miRNAs were identified, e.g., miR-1290 was upregulated in CRSwNP but downregulated in CRSsNP. Shared miRNAs across subgroups suggest core regulatory roles in CRS	Highlights miR-125b-5p as a regulator in both epithelial and peripheral immune cell functions, linking epithelial and systemic immune responses in CRS. Subgroup-specific miRNAs provide insights into phenotypic differences in CRS, aiding in patient stratification. miR-126-3p’s regulation of Th2 function and miR-708-5p’s involvement in airway inflammation suggest therapeutic potential in targeting immune dysregulation
Bu et al., 2021 [46]	Whole-transcriptome sequencing of nasal tissues from ECRSwNP (n = 10), non-ECRSwNP (n = 5), and controls (n = 9); integrated analysis of miRNA and mRNA expression; real-time PCR validation of target genes and miRNAs	Identified 3884 DE-mRNAs in ECRSwNP vs. control, 5009 DE-mRNAs in non-ECRSwNP vs. control, and 998 DE-mRNAs in non-ECRSwNP vs. ECRSwNP; miRNA families miR-154, miR-221, and miR-223 associated with both ECRSwNP and non-ECRSwNP; let-7 and miR-34/449 families associated with non-ECRSwNP. Pathway analysis revealed type 2 inflammation (eosinophil migration/chemotaxis) in ECRSwNP and type 1/type 3 inflammation (neutrophil activation, IFN-γ production) in non-ECRSwNP	Overall, this study highlights the potential for miRNA-mRNA networks to distinguish CRSwNP subtypes. miR-223’s association with eosinophilia and miR-221’s involvement in airway remodeling suggest subtype-specific biomarkers and therapeutic targets. The let-7 and miR-34/449 families offer insights into non-ECRSwNP pathogenesis and could guide phenotype-specific treatments
He et al. 2022 [52]	Plasma exosomal miRNAs were isolated from five patients with CRSwNP and five controls. Differential expression was assessed via miRNA sequencing and transcripts per million (TPM). KEGG and GO analyses were used for functional prediction of target genes.	Identified 159 differentially expressed exosomal miRNAs (93 upregulated, 66 downregulated). Notable pathways included axon guidance, extracellular matrix (ECM) receptor interaction, Hippo signaling, and Wnt/β-catenin signaling. Exosomal miRNAs may regulate epithelial–mesenchymal transition (EMT), mucosal remodeling, and inflammation.	Differential plasma exosomal miRNAs may serve as non-invasive biomarkers for CRSwNP diagnosis, monitoring persistence/recurrence, and treatment efficacy. Exosomes carrying engineered miRNAs could be a therapeutic avenue targeting EMT and inflammation.

Abbreviations: CRS: chronic rhinosinusitis; CRSwNP: chronic rhinosinusitis with nasal polyps; CRSsNP: chronic rhinosinusitis without nasal polyps; miRNA: MicroRNA; ceRNA: competitive endogenous RNA; lncRNA: long non-coding RNA; DE-miRNA: differentially expressed microRNA; DE-mRNA: differentially expressed messenger RNA; DE-lncRNA: differentially expressed long non-coding RNA; ECRSwNP: eosinophilic chronic rhinosinusitis with nasal polyps; non-ECRSwNP: non-eosinophilic chronic rhinosinusitis with nasal polyps; RT-qPCR: reverse transcription quantitative polymerase chain reaction; TPM: transcripts per million; KEGG: Kyoto Encyclopedia of Genes and Genomes; GO: gene ontology; EMT: epithelial–mesenchymal Transition; ECM: extracellular matrix; Th2: type 2 helper T-cell; IRF4: interferon regulatory factor 4; MIAT: myocardial infarction associated transcript; MSC-AS1: mesenchymal stem cell antisense RNA 1; CFI: complement factor I; AUC: area under the curve; NF-κB: nuclear factor kappa-light-chain-enhancer of activated B cells.

## 6. miRNAs as Potential Biomarkers

CRSwNP is characterized by significant heterogeneity in its underlying pathophysiology, necessitating reliable biomarkers for disease monitoring and patient stratification. Simple biomarkers like eosinophil counts and total IgE levels are widely utilized in clinical practice due to their accessibility and their association with type 2 inflammation. However, these traditional biomarkers often lack the specificity required to address the diverse inflammatory endotypes of CRSwNP, particularly in the era of biologic therapies that demand a more personalized approach [53].

Cytokines such as IL-4, IL-5, and IL-13, hallmarks of Th2 inflammation, have emerged as valuable indicators for categorizing endotypes [54]. Despite their importance in understanding disease mechanisms, these cytokines are often present at low levels in nasal tissues and secretions, limiting their practicality as routine biomarkers. In contrast, molecules like periostin (POSTN), cystatin SN (CST1), and eotaxin-3 (CCL26) have shown greater promise due to their detectability and specificity for type 2 inflammation, making them suitable for endotype-based diagnostic and therapeutic strategies [55,56]. Metalloproteinases, including ADAM8, MMP-9, and MMP-12, which play pivotal roles in tissue remodeling, are also emerging as biomarkers with the potential to reflect disease activity and progression [57,58,59]

Beyond these traditional and molecular biomarkers, non-invasive biomarkers such as exosomal miRNAs are gaining traction due to their stable presence in body fluids and hold significant potential as diagnostic and prognostic tools [53]. For instance, miR-200a-3p has been identified as a potential biomarker for CRSwNP, with its expression inversely correlating with disease severity, reflecting its dual role in diagnosing CRSwNP and monitoring disease progression [26]. Similarly, miR-205-5p is significantly upregulated in CRSwNP patients and is associated with more severe Th2 inflammation, higher eosinophil counts, worse SNOT-22 scores, and poor clinical outcomes [33]. MiR-125b has also emerged as a promising biomarker, particularly for predicting outcomes following endoscopic sinus surgery (ESS). Elevated miR-125b levels in nasal polyps have been strongly linked to poorer postoperative results and thus could enable clinicians to identify high-risk patients and tailor postoperative management strategies, such as closer monitoring or adjunctive therapies [60].

Recent therapeutic advances further highlight the utility of miRNAs as biomarkers. A study exploring the effects of dupilumab, an add-on biologic therapy for CRSwNP, found that miR-25-3p and miR-185-5p were downregulated following treatment [61]. These miRNAs have been proposed as non-invasive biomarkers for monitoring treatment response and long-term prognosis in patients undergoing dupilumab therapy.

Biomarkers for CRSwNP are evolving from traditional measures such as eosinophil counts and IgE levels to more specific molecular and non-invasive tools. Among these, miRNAs represent a transformative class of biomarkers due to their stability, specificity, and versatility in reflecting disease characteristics and treatment responses. Continued research into miRNA expression profiles and their functional roles will be instrumental in advancing personalized medicine approaches for CRSwNP, improving diagnostic accuracy, disease monitoring, and therapeutic outcomes.

## 7. MiRNAs as Potential Therapeutic Targets

CRSwNP is a complex inflammatory disease that often necessitates a multifaceted therapeutic approach. While surgery can reduce the disease burden by removing polyps, it does not address the underlying inflammatory processes, making recurrence a common issue. To achieve more durable disease control, novel biological therapies targeting specific inflammatory pathways have been developed. Currently, monoclonal antibodies such as dupilumab (anti-IL-4Rα), omalizumab (anti-IgE), mepolizumab (anti-IL-5), and benralizumab (anti-IL-5R) are approved for CRSwNP treatment, particularly in cases of the inadequately controlled disease. Despite their promise, not all patients respond to these therapies, highlighting the heterogeneity within the type 2 endotype of CRSwNP [55]. Moreover, the high cost of biologics, ranging from USD 10,000 to USD 40,000 annually, underscores the need for predictive biomarkers to identify responders and improve treatment efficiency [62].

**Figure 2:**
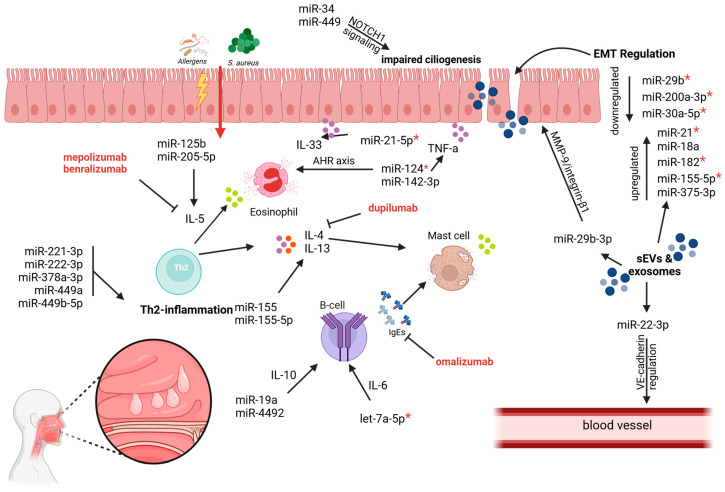
MicroRNAs modulating inflammation and tissue remodeling in CRSwNP. MiRNAs regulate epithelial–mesenchymal transition (EMT), vascular permeability, and inflammation in CRSwNP. MiR-22-3p downregulates VE-cadherin, contributing to edema, while miR-29b-3p promotes polyp formation via MMP-9-integrin β1 complexes. Downregulated miR-34 and miR-449 impair ciliogenesis by failing to suppress NOTCH1. Small extracellular vesicles (sEVs) and exosomes facilitate miRNA transport, emphasizing their role in intracellular communication and CRSwNP pathogenesis. Pro-inflammatory cytokines IL-4, IL-5, IL-13, and TNF-α are elevated by miR-155, miR-205-5p, miR-21-5p, miR-125b, and miR-142-3p, fueling Th2-dominant inflammation. MiRNAs marked with * demonstrate potential for reducing inflammation and nasal polyp formation through the use of mimics or inhibitors to reverse their regulation. Currently approved monoclonal antibodies, including dupilumab (anti-IL-4Rα/IL013Ra), omalizumab (anti-IgE), mepolizumab (anti-IL-5), and benralizumab (anti-IL-5R), for CRSwNP treatment, targeting inflammatory pathways. Created in BioRender.

As personalized medicine continues to evolve, the integration of targeted biologic therapies and predictive biomarkers offers hope for more effective and tailored management of CRSwNP. Among these advancements, microRNAs (miRNAs) have emerged as promising therapeutic targets due to their capacity to regulate key inflammatory and remodeling pathways (Figure 2).

### 7.1. Targeting EMT

The role of EMT in CRSwNP pathophysiology is well established, with miRNAs serving as key regulators of this process. For instance, miR-182 inhibition reverses TGF-β1-induced EMT, reducing inflammation and tissue remodeling in nasal polyps (18). Similarly, Cyclin-dependent kinase 6 (CDK6) acts as a regulator targeting miR-30a-5p to inhibit EMT, further underscoring the therapeutic potential of modulating miRNA expression [24]. Inhibitors of miR-155-5p reverse TGF-β1-induced EMT by preserving SIRT1 expression, leading to reduced mesenchymal markers and increased epithelial markers [22]. Similarly, miR-29b mimics and siHSP47 effectively target the TGF-β1/miR-29b/HSP47 axis, reversing EMT and reducing tissue remodeling [21].

### 7.2. Inflammatory Pathways and Immune Modulation

MiRNAs also regulate inflammatory pathways central to CRSwNP. MiR-21-5p inhibition suppresses inflammation through the miR-21-5p/GLP1R/IL-33 axis, reducing eosinophilic infiltration and Th2 cytokine levels, including IL-4, IL-5, and IL-13 [30]. Let-7a-5p mimics reduce pro-inflammatory cytokines such as TNF-α, IL-1β, and IL-6 while inhibiting the Ras-MAPK signaling pathway, highlighting its dual anti-inflammatory and anti-remodeling potential. However, as IL-6 can counteract let-7a-5p’s effects, a combined therapeutic approach targeting both pathways may be necessary [31].

### 7.3. Emerging Therapeutic Pathways

Other miRNAs target broader inflammatory and remodeling pathways. The miR-200a-3p/ZEB1/ERK-p38 axis represents a pivotal pathway in CRSwNP. MiR-200a-3p mimics or ZEB1 inhibitors restore epithelial integrity, suppress EMT markers, and reduce pro-inflammatory cytokines such as IL-4, IL-5, and IL-13 [26]. The EGF/HIF-1α/miR-21 axis is another promising target. Interventions such as EGF inhibition using short hairpin RNA (sh-EGF) and anti-miR-21 oligonucleotides suppress inflammation and epithelial remodeling, offering a novel strategy for managing CRSwNP [19]. Additionally, enhancing miR-124 levels or inhibiting AHR via siRNA has demonstrated efficacy in reducing inflammatory responses, highlighting the potential of the miR-124/AHR axis as a therapeutic target [37].

### 7.4. The Role of Small Extracellular Vesicles (sEVs) and Exosomes

Small extracellular vesicles (sEVs), including exosomes—a subset of sEVs—play a crucial role in transferring miRNAs like miR-375-3p, which target epithelial–mesenchymal transition (EMT) and inflammatory pathways, contributing to the pathogenesis of CRSwNP [23]. By leveraging their natural ability to deliver cargo to specific cells, engineered sEVs and exosomes can be designed to carry miRNA mimics or inhibitors, enabling precise modulation of disease-related pathways. This approach not only holds promise for effectively targeting EMT and inflammation but also offers the potential to minimize systemic side effects, advancing the therapeutic landscape of CRSwNP [63].

## 8. Current Gaps and Future Research Directions

Our review of the viability and application of miRNAs as epigenetic regulators of CRSwNP unveiled several critical research gaps that should be outlined to provide directions for future research. One notable limitation is the lack of diversity in study populations. Most existing research has been conducted in Asia, which restricts the generalizability of findings. Expanding studies to include racially and geographically diverse populations is crucial for identifying variations in epigenetic mechanisms across different demographics. Another area requiring attention is the influence of environmental factors such as microbial exposure, smoking, and air pollution on epigenetic changes in CRSwNP [4]. These factors remain underexplored, yet they may play a significant role in disease development and progression. Longitudinal studies examining the impact of these environmental stimuli could provide valuable insights.

The interpretation of epigenetic data in general is further complicated by the presence of comorbid conditions, including asthma and allergic rhinitis. Asthma, for example, shares overlapping inflammatory pathways and immune responses with CRSwNP, potentially confounding findings specific to the latter. Similarly, allergic rhinitis can interact with CRSwNP-associated inflammation, making it difficult to isolate epigenetic changes unique to the disease [15]. Future research should carefully account for these comorbidities to ensure clarity in identifying CRSwNP-specific epigenetic mechanisms.

Additionally, existing transcriptomic datasets from CRS studies could be utilized to identify and compare non-coding RNA profiles across diverse populations. This approach has the potential to yield valuable insights but must be conducted with rigorous matching of disease and control tissues to ensure validity. Consistency in bioinformatic analyses is also essential. Ensuring that studies use comparable methodologies and tissue sources will improve the reliability of findings and enable meaningful comparisons across studies. There is a lack of consistency in miRNA quantification methods, normalization protocols, and reproducibility between studies, making it difficult to generalize findings. On this account, it should be noted that this review exclusively utilized PubMed for the literature search due to its clinical focus. While this ensured specificity and relevance, the inclusion of additional databases could provide broader coverage of the topic and should be considered in future work.

A key direction for future research is the development of personalized treatment strategies based on individual patient characteristics. CRSwNP is a complex inflammatory respiratory disease involving multiple cell types, including nasal epithelial cells, dendritic cells, eosinophils, mast cells, macrophages, and Th2 cells. Epigenetic profiling, based on miRNA expression patterns in some or all of these cells, could be used to stratify patients into subgroups with distinct disease endotypes [46]. Gene therapy could then be applied accordingly. CRISPR-Cas9 and other gene-editing technologies have shown promise in modulating miRNA expression in disease settings, highlighting their possible application in CRSwNP to regulate inflammation and tissue remodeling [64].

Additionally, RNA-based therapies such as mimics and antagomirs, hold significant promise for modulating disease-associated and disease-subtype-specific miRNAs. These therapies could be delivered locally to the nasal mucosa using nanoparticle-based delivery systems, minimizing systemic side effects. However, this is easier said than done, and we are currently far from achieving this goal. Nonetheless, research is ongoing to decipher the logistics of such approaches. For example, studies on RNA-binding proteins (RBPs) in miRNA transport to exosomes offer a novel angle for therapeutic development [23]. Similarly, researchers can potentially design targeted therapies that use exosomal miRNA pathways to regulate inflammatory progression and airway remodeling in CRSwNP [63]. However, preclinical studies are essential to evaluate the safety and efficacy of these approaches, followed by clinical trials in well-defined patient cohorts.

## 9. Conclusions

In summary, miRNAs play an important role in the pathogenesis of CRSwNP, regulating inflammation and tissue remodeling. First, they have emerged as promising biomarkers, offering the potential for assessing disease progression and predicting treatment response. Second, miRNA profiling has provided deeper insight into patient stratification, allowing for the identification of distinct disease endotypes that may guide more targeted therapeutic interventions. Third, therapeutic modulation of miRNAs represents a promising avenue for novel treatment strategies in CRSwNP, with exosomes serving both as non-invasive biomarkers and as potential delivery vehicles for targeted therapies.

Despite these advancements, challenges remain in standardizing methodologies and ensuring reproducibility across studies, which is crucial for translating findings into clinical applications. Continued research and methodological refinement will be essential to fully integrate miRNA-based strategies into personalized medicine for CRSwNP.

## Figures and Tables

**Table 1:** MiRNAs associated with inflammatory pathways in CRSwNP.

MicroRNA	Expression Status	Target Genes/Pathways	Functional Role in CRSwNP	Reference(s)
miR-18a	upregulated	PTEN regulation, activation of PI3K/AKT pathway	contributes to epithelial–mesenchymal transition (EMT)	Jian and Yin, 2022 [25]
miR-21	upregulated	PDCD4, NF-κB, PTEN, Akt	facilitates EMT via the TGF-β1-miR-21-PTEN-Akt axis; exhibits anti-inflammatory effects through IL-10 regulation	Li, 2019 [20] Liu, 2021 [29]
		EGF/HIF-1α/miR-21/AQP4 axis	Promotes epithelial cell proliferation and survival, promotes EMT; contributes to inflammation and tissue remodeling	Chen et al., 2022 [19]
miR-125b	upregulated	Wnt/β-catenin signaling pathway	increased inflammation and fibrin deposition contributing to NP development	Song et al., 2022 [44]
	upregulated	IFN signaling pathway, elevating IL-5	Leads to eosinophil infiltration and inflammation in eosinophilic CRSwNP	Gata et al., 2023 [27] Zhang et al., 2012 [28]
miR-142-3p	upregulated	TNF-α, LPS-TLR signaling	drives inflammatory responses by amplifying the LPS-TLR-TNF-α pathway	Qing et al., 2021 [38]
miR-182	upregulated	TGF-β1 induced EMT pathways	regulation of EMT in response to inflammation	Jiang et al., 2024 [18]
miR-30a-5p	downregulated	TGF-β1 induced EMT pathways	promotes EMT in nasal polyps	Zhang et al., 2021 [24]
miR-19a	upregulated	IL-10	suppresses IL-10 production, exacerbating inflammation and immune dysregulation	Luo, 2017 [34]
miR-155	upregulated	NF-κB pathway, upregulated COX2, downregulated SOCS1	expression of inflammatory cytokines TNF-α, IL-1, IL-4, and IL-5 amplifies inflammation	Du et al., 2020 [32]
miR-155-5p	upregulated	SIRT1, TP53INP1	promotes EMT and Th2 cytokine secretion and ILC2 survival, aggravating inflammation	Zhu, 2021 [45] Yang, 2020 [22]
miR-29b-3p	downregulated	MMP-9, integrin β1	contributes to polyp formation through α-tubulin deacetylation and increased matrix metalloproteinase activity	Liu, 2021 [42]
miR-22-3p	upregulated	VE-cadherin	elevates vascular permeability, fostering tissue edema, and nasal polyp growth	Zhang, 2020 [39]
miR-150-5p	upregulated	EGR2	elevated IL-17, activation and proliferation of CD4+ T cells into Th17, dysregulation of dendritic cells (DC) leading to inflammation in CRS	Ma et al., 2018 [35]
miR-214	upregulated	STAT3/GDF15 pathway and SIRT1	regulates LPS-mediated inflammation and MUC5AC expression	Whang et al., 2023 [41]
miR-375-3p	upregulated	miR-375-3p in sEVs inhibits KH domain containing RNA binding (QKI)	Promotes EMT by suppressing QKI	Wang et al., 2024 [23]
miR-4492	downregulated	miR-4492/IL-10 interaction in the Jak/STAT signaling pathway	Inflammation and tissue remodeling in nasal polyps	Li et al., 2019 [36]
miR-34	downregulated	NOTCH1 signaling pathway and reduced expression of ciliogenesis regulators MYB, CCNO, FOXJ1	Impaired ciliogenesis and defective cilia structure leads to impaired mucociliary clearance in the epithelium of CRSwNP	Callejas et al., 2020 [43]
miR-449	downregulated			
miR-200a-3p	downregulated	ZEB1, ERK/p38 signaling pathway, MAPK signaling pathway	suppresses EMT and inflammation by regulating ZEB1; protects nasal epithelial cells from tissue remodeling	Wu et al., 2024 [26]
miR-205-5p	upregulated	promotes IL-5 and eosinophilia	enhances T2 inflammation	Silveira et al., 2021 [33]
miR-221-3p	upregulated	Implicated in cell cycle regulation and apoptosis	Implicated to inflammation in a minor extend	
miR-222-3p				
miR-378a-3p				
miR-449a				
miR-449b-5p				
miR-21-5p	upregulated	Glucagon-like peptide-1 receptor (GLP-1R)/IL-33 signaling	aggravates Th2-inflammation by promoting eosinophil infiltration and IL-33 expression	Luan et al., 2022 [30]
let-7a-5p	downregulated	IL-6, Ras-MAPK signaling pathway	Negatively regulates TNF-α, IL-1β, and IL-6 expression; reduces inflammation by inhibiting Ras-MAPK signaling pathway	Zhang et al., 2021 [31]
miR-124	downregulated	Negative regulation of aryl hydrocarbon receptor (AHR) expression, and TNF-a	reduces inflammation by inhibiting AHR-mediated inflammatory signaling	Liu et al., 2018 [37]
miR-29b	downregulated	TGF-β1/miR-29b/HSP47 axis	promotes EMT and tissue remodeling	Shin et al., 2021 [21]

Abbreviations: CRSwNP: chronic rhinosinusitis with nasal polyps; CRS: chronic rhinosinusitis; EMT: epithelial–mesenchymal transition; miRNA: MicroRNA; PTEN: phosphatase and tensin homolog; PI3K/AKT: phosphoinositide 3-kinase/protein kinase B signaling pathway; NF-κB: nuclear factor kappa-light-chain-enhancer of activated B cells; SOCS1: suppressor of cytokine signaling 1; SIRT1: sirtuin 1; MMP-9: matrix metalloproteinase 9; VE-cadherin: vascular endothelial cadherin; IL: interleukin; TNF-α: tumor necrosis factor alpha; ERK: extracellular signal-regulated kinase; MAPK: mitogen-activated protein kinase; AHR: aryl hydrocarbon receptor; TGF-β1: transforming growth factor beta 1.

## Abbreviations

The following abbreviations are used in this manuscript:

CRSwNP	chronic rhinosinusitis with nasal polyps
miRNAs	microRNAs
EMT	epithelial–mesenchymal transition
sEVs	small extracellular vesicles
CRS	chronic rhinosinusitis
Th2	type 2 helper T-cell
IL	interleukin
COPD	chronic obstructive pulmonary disease
HDAC	histone deacetylase
APA	alternative polyadenylation
UTR	3’ untranslated region
ncRNAs	non-coding RNAs
TGF-β1	transforming growth factor beta 1
PTEN	phosphatase and tensin homolog
PI3K/AKT	phosphoinositide 3-kinase/protein kinase B signaling pathway
NF-κB	nuclear factor kappa-light-chain-enhancer of activated B cells
COX2	cyclooxygenase-2
SOCS1	suppressor of cytokine signaling 1
SIRT1	sirtuin 1
TP53INP1	tumor protein P53 inducible nuclear protein 1
MMP-9	matrix metalloproteinase 9
VE-Cadherin	vascular endothelial cadherin
EGR2	early growth response protein 2
STAT3/GDF15	signal transducer and activator of transcription 3/growth differentiation Factor 15
QKI	quaking protein
NOTCH1	notch receptor 1
MYB	myeloblastosis transcription factor
CCNO	Cyclin O
FOXJ1	forkhead box protein J1
ZEB1	zinc finger E-box binding homeobox 1
ERK	extracellular signal-regulated kinase
MAPK	mitogen-activated protein kinase
GLP-1R	glucagon-like peptide-1 receptor
AHR	aryl hydrocarbon receptor
HSP47	heat shock protein 47
T2	type 2 inflammation
TNF-α	tumor necrosis factor alpha
LPS	lipopolysaccharide
Sp1	specificity Protein 1
ceRNA	competitive endogenous RNA
DCs	dendritic cells
lncRNAs	long non-coding RNAs
MIAT	myocardial infarction associated transcript
IRF4	interferon regulatory factor 4
CFI	complement factor I
MSC-AS1	mesenchymal stem cell antisense RNA 1
CRSsNP	chronic rhinosinusitis without nasal polyps
DE-miRNA	differentially expressed microRNA
DE-mRNA	differentially expressed messenger RNA
DE-lncRNA	differentially expressed long non-coding RNA
ECRSwNP	eosinophilic chronic rhinosinusitis with nasal polyps
non-ECRSwNP	non-eosinophilic chronic rhinosinusitis with nasal polyps
RT-qPCR	reverse transcription quantitative polymerase chain reaction
TPM	transcripts per million
KEGG	Kyoto Encyclopedia of Genes and Genomes
GO	Gene ontology
ECM	extracellular matrix
